# Supplementary material for: HIVprotI: an integrated web based platform for prediction and design of HIV proteins inhibitors
Source: J Cheminform. 2018 Mar 9;10:12. doi: 10.1186/s13321-018-0266-y (PMC5845081; doi:10.1186/s13321-018-0266-y)
Supplement: Supplementary file 2 — Additional file 2. Source code of HIVProtI web server. [file 13321_2018_266_MOESM2_ESM.zip › HIVprotI_Source-code/hivprotI/weka-3-6-6/doc/weka/gui/beans/FlowRunner.SimpleLogger.html]

FlowRunner.SimpleLogger


---


|  |  |  |
| --- | --- | --- |
| **Overview** \| **Package** \| **Class** \| **Tree** \| **Deprecated** \| **Index** \| **Help** \| **Weka's home** | |  |
| **PREV CLASS**   **NEXT CLASS** | **FRAMES**    **NO FRAMES**     **All Classes** |  |
| SUMMARY: NESTED \| FIELD \| CONSTR \| METHOD | DETAIL: FIELD \| CONSTR \| METHOD |  |


---


## weka.gui.beans Class FlowRunner.SimpleLogger

```
java.lang.Object
  weka.gui.beans.FlowRunner.SimpleLogger
```

**All Implemented Interfaces:**: Logger

**Enclosing class:**: FlowRunner

---

``` public static class FlowRunner.SimpleLogger extends java.lang.Object implements Logger ```

---

**Constructor Summary**

`FlowRunner.SimpleLogger()`


| **Method Summary** | |
| --- | --- |
| `void` | `logMessage(java.lang.String lm)`             Sends the supplied message to the log area. |
| `void` | `statusMessage(java.lang.String lm)`             Sends the supplied message to the status line. |

**Methods inherited from class java.lang.Object**

`equals, getClass, hashCode, notify, notifyAll, toString, wait, wait, wait`

**Constructor Detail**

### FlowRunner.SimpleLogger

```
public FlowRunner.SimpleLogger()
```


**Method Detail**

### logMessage

```
public void logMessage(java.lang.String lm)
```

:   **Description copied from interface: `Logger`**
:   Sends the supplied message to the log area. These message will typically
    have the current timestamp prepended, and be viewable as a history.

    :   **Specified by:**: `logMessage` in interface `Logger`
    :   **Parameters:**: `lm` - the log message

---


### statusMessage

```
public void statusMessage(java.lang.String lm)
```

:   **Description copied from interface: `Logger`**
:   Sends the supplied message to the status line. These messages are
    typically one-line status messages to inform the user of progress
    during processing (i.e. it doesn't matter if the user doesn't happen
    to look at each message)

    :   **Specified by:**: `statusMessage` in interface `Logger`
    :   **Parameters:**: `lm` - the status message.


---


|  |  |  |
| --- | --- | --- |
| **Overview** \| **Package** \| **Class** \| **Tree** \| **Deprecated** \| **Index** \| **Help** \| **Weka's home** | |  |
| **PREV CLASS**   **NEXT CLASS** | **FRAMES**    **NO FRAMES**     **All Classes** |  |
| SUMMARY: NESTED \| FIELD \| CONSTR \| METHOD | DETAIL: FIELD \| CONSTR \| METHOD |  |


---
